# Ginkgo Biloba Leaf Extract Improves an Innate Immune Response of Peripheral Blood Leukocytes of Alzheimer’s Disease Patients

**DOI:** 10.3390/nu14102022

**Published:** 2022-05-11

**Authors:** Marta Sochocka, Michał Ochnik, Maciej Sobczyński, Katarzyna Gębura, Aleksandra Zambrowicz, Piotr Naporowski, Jerzy Leszek

**Affiliations:** 1 Laboratory of Virology, Department of Immunology of Infectious Diseases, Hirszfeld Institute of Immunology and Experimental Therapy, Polish Academy of Sciences, 58-114 Wroclaw, Poland; michal.ochnik@hirszfeld.pl; 2 Laboratory of Molecular Neurobiology, Nencki Institute of Experimental Biology of the Polish Academy of Sciences, 02-093 Warsaw, Poland; macsebsob@poczta.onet.pl; 3 Laboratory of Clinical Immunogenetics and Pharmacogenetics, Hirszfeld Institute of Immunology and Experimental Therapy, Polish Academy of Sciences, 53-114 Wroclaw, Poland; katarzyna.gebura@hirszfeld.pl; 4 Department of Animal Products Technology and Quality Management, Faculty of Biotechnology and Food Sciences, Wroclaw University of Environmental and Life Sciences, 51-630 Wroclaw, Poland; aleksandra.zambrowicz@upwr.edu.pl; 5 Laboratory of Medical Microbiology, Department of Immunology of Infectious Diseases, Hirszfeld Institute of Immunology and Experimental Therapy, Polish Academy of Sciences, 53-114 Wroclaw, Poland; piotr.naporowski@hirszfeld.pl; 6 Department of Psychiatry, Wroclaw Medical University, 50-367 Wroclaw, Poland; jerzy.leszek@umed.wroc.pl

**Keywords:** extract of *Ginkgo biloba* (EGb), innate immunity, PBLs, Alzheimer’s disease, cytokines

## Abstract

Background: One of the main features of Alzheimer’s disease (AD) pathology is failure in innate immune response and chronic inflammation. Lack of effective AD treatment means that more attention is paid to alternative therapy and drugs of natural origin, such as extract of *Ginkgo biloba* (EGb). The purpose of this study was to investigate the effect of EGb on the mechanisms of innate immune response of peripheral blood leukocytes (PBLs) in AD patients. Methods: In AD patients and healthy-age matched controls, the effect of EGb on two of innate immune reactions, i.e., PBLs resistance to viral infection ex vivo and production of cytokines, namely TNF-α, IFN-γ, IL-1β, IL-10, IL-15, and IFN-α, were investigated. The influence of EGb on inflammatory-associated genes expression that regulate innate immune response to viral infection and cytokine production, namely *IRF-3, IRF-7, tetherin, SOCS1, SOCS3, NFKB1, p65,* and *MxA* was also examined. Results: A beneficial effect of EGb especially in AD women was observed. EGb decreased production of TNF-α, IFN-γ, and IL-10 and increased IL-15 and IL-1β. The effect was more pronouncement in AD group. EGb also downregulated expression of investigated genes. Conclusions: EGb may have an advantageous properties for health management in elderly and AD sufferers but especially in women with AD. Improving peripheral innate immune cells’ activity by adding EGb as accompanying treatment in AD may be, in the long term, a good course to modify the disease progression.

## 1. Introduction

Immunity and chronic inflammation play a key role in the survival of the older adults, and according to the latest knowledge, they also represent one of the main features of Alzheimer’s disease (AD) pathology [1]. AD is one of the most important age-related health problems worldwide, and it is believed that the onset and progression of the disease may depend at least in part on optimal immune system functioning. Experimental studies highlight the pathological changes in the central and peripheral immune response in AD [2,3]. An increased levels of peripheral inflammatory markers, such as IL-6, TNF-α, or C-reactive protein (CRP), were found to be associated with future cognitive decline and dementia [2,4]. Currently, no effective drugs are available for the treatment of AD symptoms. An accessible pharmacotherapy aims to only slow disease progression and reduce cognitive symptoms. Some hope is associated with GV-971 (sodium oligomannate capsules), which improved cognitive functions in AD patients in China in a phase 3 trial and was approved for the treatment of AD in China [5]. Thus, more attention is paid to alternative therapy, such as using drugs of natural origin. Moreover, searching for natural compounds with immunoregulatory activity seems to be a good direction for future adjunct AD therapy.

Currently, phytomedicine is gaining its popularity, and many plant-derived phytotherapeutics with medicinal properties are used in the treatment of various diseases, including age-related diseases [6]. The phytomedicine of aging provide a wide range of bioactive compounds, such as flavonoids, terpenoids, or polyphenols with therapeutic effects. Health benefits consist mainly of acting as an immunity booster and exhibiting antioxidant, cardio-protective, and neuro-protective effects [7]. One of the most popular medicinal plants is *Ginkgo biloba*. Standardized extract of *G. biloba* (EGb) contains 24% ginkgo flavonoid glycosides, 6% terpene lactones, and up to 5 ppm ginkgolic acids [8]. The therapeutic potential of EGb is manifested in beneficial effect on the circulatory system (blood flow improvement, prevention of clot formation, reinforcing the walls of the capillaries) and nervous system with protection of nerve cells from injury [9]. Phytochemical constituents from *G. biloba,* such as flavonoids and terpenoids, showed beneficial effect in the treatment of concentration difficulties, memory impairment, and AD. Thus, EGb is considered as memory enhancer [10].

The use of phytotherapeutics/nutraceuticals as an adjunct therapy to classic drug therapies is highly recommended in many diseases. However, many more studies are still needed to evaluate the therapeutic potential and clarifying the mechanism of action of natural compounds, including EGb. It is believed that this could help to choose better phytotherapeutics as the accompanied treatment of neurodegenerative pathologies such as AD. EGb is already used in the treatment of AD and cognitive deficits acting as anti-aggregating and pro-cognitive preparation. It is implemented to improve memory impairment and cognitive decline [11]. However, less attention and research are concentrated on its effect on immune system functioning in AD patients. The purpose of this study was to investigate the effect of EGb on the mechanisms of innate immune response of peripheral blood leukocytes (PBLs) of AD sufferers.

## 2. Materials and Methods

### 2.1. Blood Samples

Peripheral venous blood was obtained from 39 Subjects: 22 AD patients (15 females, 7 males) and 17 healthy adult volunteers (10 females, 7 males) of an appropriate age (43–90 years) and collected in tubes containing anticoagulant EDTA or heparin. Patients were under the care of Department of Psychiatry of the Medical University in Wroclaw, Poland. Patients did not receive any anti-dementia and other drugs before blood venipuncture as well as any other immunomodulators. Among the patients, no infectious diseases occurred in the 3-month period before the inclusion to the study.

### 2.2. Ethics Approval and Consent to Participate

This study has been reviewed, approved, and conducted in accordance with the guidelines of the Ethics Committee of the Wroclaw Medical University (No. KB-349/2016). Signed consent was obtained from all participants of the study or their legal representative.

### 2.3. Clinical Examination

Patients were under psychiatric and neurological examinations as well as laboratory tests, electroencephalographic examinations (EEG), and computer tomography (CT) or magnetic resonance imaging (MRI) structural studies. Mini-Mental State Examination (MMSE) was used for the screening of dementia. All patients met DSM-V and NINCDA-ADRDA criteria for probable AD dementia. A diagnosis of AD was made when specific symptoms were present and by making sure other causes of dementia were absent, including anemia, brain tumor, chronic infection, intoxication from medication, severe depression, stroke, thyroid disease, and vitamin deficiencies. CT and MRI of the brain were performed as well to look for other causes of dementia, such as brain tumor or stroke. Semi-structured interview with the patient and informant, physical exam, evaluation of neurological status, and psychiatric exam were obtained. Vital signs and blood screening labs (hematology, chemistry panel, urinalysis, vitamin B12 (B12), thyrotropin (TSH)) were collected. *Exclusion criteria:* patients older than 90 years, any significant neurological disease such as Parkinson’s disease, multi-infarct dementia, Huntington disease, normal pressure hydrocephalus, brain tumor, progressive supranuclear palsy, seizure disorder, subdural hematoma, multiple sclerosis, or history of significant head trauma followed by persistent neurologic defaults or known structural brain abnormalities. MRI scan with evidence of infection, infarction, or other focal lesions; subjects with multiple lacunes or lacunes in a critical memory structure; psychiatric disorder/psychotic features: major depression, bipolar disorder, agitation, or behavioral problems within the last 3 months; history of schizophrenia, alcohol abuse, history of alcohol or substance abuse or dependence within the past 2 years; any significant systemic illness or unstable medical condition; clinically significant abnormalities in B12, rapid plasma regain test (RPR), or TSH; and current use of specific psychoactive medications (e.g., certain antidepressants, neuroleptics, chronic anxiolytics, or sedative hypnotics, etc.). Patients were excluded if they did not agree to respond to the test questions and/or if they had life-threatening diseases other than AD.

### 2.4. Extract of G. Biloba (EGb)

Standardized dry extract from *G. biloba* leaves (GINKGONIS EXTRACTUM SICCUM RAFFINATUM ET QUANTIFICATUM PH. EUR. (European Pharmacopoeia)) provided by Martin Bauer Group, Finzelberg GmbH & Co. KG, Andernach, Germany, was investigated. EGb is a dry extract from *G. biloba* leaves. The extract is adjusted to 22.0–27.0% ginkgo flavonoids calculated as ginkgo flavone glycosides and 5.0–7.0% terpene lactones consisting of 2.8–3.4% ginkgolides A, B, and C and 2.6–3.2% bilobalide and contains less than 5 ppm ginkgolic acids.

*EGb solution:* Before each experiment, EGb was dissolved in dimethyl sulfoxide (DMSO) at a primary concentration of 20 mg/mL and mixed thoroughly until complete dissolution. Next, EGb solution in DMEM 2% FBS medium was prepared for experiments with PBLs. For antioxidant activity powder of EGb was dissolved in 96% ethanol (1 mg/mL).

### 2.5. Trypan Blue Staining for Cell Viability

The viability of PBLs was measured with 0.4% trypan blue staining. A total of 100 µL of cell suspension (1 × 10^6^ cells/mL) was incubated with 100 µL of 0.4% trypan blue. After 15 min of incubation at room temperature, the viability of the cells was measured in a Bürker chamber with the use of light microscope (Olympus CX31). Dead cells were labeled with navy-blue, and live cells remained unstained.

### 2.6. Determination of Antioxidant Activity as the Ability to Scavenge DPPH Free Radicals

The antioxidant activity of the obtained hydrolysates was assessed on the basis of the radical scavenging effect of the stable 1,1-diphenyl-2-picrylhydrazyl (DPPH (Sigma, St. Louis, MO, USA, D21140-0)) free radical activity according to Yen and Chen with minor modifications [12]. The tested samples were dissolved in water to a final volume of 1 mL and mixed with 1 mL of ethanol (98%). The reaction was started by adding 0.5 mL of 0.3 M DPPH in ethanol. The mixtures were left for 30 min at room temperature, and the absorbance of the resulting solutions was measured at 517 nm. For calibration, aqueous solutions of known Trolox concentrations ranging from 2 to 20 μg (able to scavenge 500 μL of 0.3 mM DPPH radical solution) were used. Radical scavenging activity of the peptides was expressed as µM Trolox_eq_/mg protein.

### 2.7. FRAP Method

The FRAP method (ferric-reducing antioxidant power) was used to determine the antioxidative capacity of hydrolysates according to Benzie and Strain [13]. A total of 3 mL of FRAP working solution (300 mM acetate buffer pH 3.6; 10 mM 2,4,6,tripyridyl-s-triazine (TPTZ) (Fluka, 93285) and 20 mM FeCl_3_ × 6 H_2_O (10:1:1 *v*/*v*)) was mixed with 1 mL of the sample. After 10 min of reaction, the absorbance was measured at λ = 593 nm. An aqueous solution of known Fe (II) concentration was used for calibration (in the range from 100 to 1000 μg). Results were expressed as μg Fe^2+^/mg protein.

### 2.8. Determination of Fe (II) Ion Chelation

Chelation of iron ions by hydrolysates was estimated by the method of Xu et al. [14] with modifications. A 250 μL sample was mixed with 1250 μL H_2_O and 110 μL 1 mM FeCl_2_. After 2 min, 1 mL of 500 μM ferrozine (Sigma, 160601) aqueous solution was added and the mixture was allowed to react for 10 min. The absorbance of ferrous iron–ferrozine complex was measured spectrophotometrically at λ = 562 nm. A known concentration of FeCl_2_ (0–20 μg) was used to generate a standard curve, and the ability to chelate iron ions was expressed as μg Fe^2+^/mg protein.

### 2.9. Virus and Cell Line

A wild-type Indiana VSV (*Vesicular stomatitis virus, Rhabdoviridae*) serotype was used. VSV was obtained from Dr. C. Buckler (National Institutes of Health, Bethesda, MD, USA). Virus was grown and titrated in L_929_ cells. Viral titer was expressed with reference to the TCID_50_ (tissue culture infectious dose) value, based on the cytopathic effect caused by this virus in approximately 50% of infected cells.

L_929_ (ATCC CCL1), a murine fibroblast-like cell line, was maintained in complete RPMI 1640 medium (HIIET, Wroclaw, Poland) with antibiotics (100 U/mL penicillin and 100 μg/mL streptomycin), 2 mM l-glutamine, and 2% fetal bovine serum (FBS) (all from Merck KGaA, Darmstadt, Germany).

### 2.10. Isolation of Peripheral Blood Leukocytes (PBLs)

PBLs were isolated according to a standard protocol from 10 mL of peripheral blood by gradient centrifugation in Gradisol G (Aqua-Med, Łódź, Poland) and maintained in RPMI 1640 medium (HIIET, Wroclaw, Poland) with antibiotics (100 U/mL penicillin and 100 μg/mL streptomycin), 2 mM l-glutamine, and 2% FBS (Merck KGaA, Darmstadt, Germany).

### 2.11. Determination of Resistance/Level of Innate Immunity of PBLs

Resistance/innate immunity was determined by infection of leukocytes (1 × 10^6^ cells/mL) ex vivo with a VSV dose of 100 TCID_50_. After 40 min of adsorption at room temperature (rt), the virus was washed out three times with RPMI medium with 2% FBS, and the cells were suspended in 1 mL of RPMI 2% FBS for investigations of the influence of EGb on PBLs resistance/innate immunity and cytokine production. A sample of the infected cells was kept at 4 °C and served as a control of the starting level of the virus. The rest of the cells were divided to two parts, i.e., VSV-infected and uninfected, and next were treated with 150 µg/mL EGb and incubated at 37 °C for 24 h. After that, time samples of the medium above the cells were collected and titrated in L_929_ cells. Viral titer was expressed in TCID_50_. Resistance of PBLs to VSV infection was assessed as follows: a VSV titer ≥ 4 log TCID_50_ was considered as a lack of resistance (deficiency in innate immunity), a titer of 2–3 log indicated partial resistance, and a titer of 0–1 log indicated complete resistance to VSV infection (high level of innate immunity).

### 2.12. Cytokine Measurement

The levels of IL-1β, IL-10, IL-15, IFN-α, IFN-γ, and TNF-α in supernatants from uninfected and VSV-infected leukocytes were detected using enzyme-linked immunosorbent assays (BD OptEIA TM human IL-1β, IL-10, IL-15, IFN-γ,TNF-α ELISA set, BD Biosciences; IFN-α Human ELISA Kit, Thermo Fisher, Carlsbad, CA, USA). The optical density was measured at 450 nm with λ correction 570 nm using a Multiskan RC spectrophotometric reader (Thermo Labsystems, Philadelphia, PA, USA). Cytokine concentrations were expressed in pg/mL.

### 2.13. RNA Isolation and Real-Time PCR

Real-time PCR was used to investigate mRNA expression. Total RNA (obtained from uninfected and VSV-infected cells treated with EGb 150 µg/mL) was extracted with the Relia Prep™ RNA Cell Miniprep System kit (Promega, Madison, WI, USA), and reverse transcription was performed using the High Capacity cDNA Reverse Transcription kit (Applied Biosystem, Thermo-fisher Scientific, Carlsbad, CA, USA) according to the manufacturer’s instructions. Reaction was performed in T3000 Thermocycler (Biometra, Göttingen, Germany). Reaction volume was 20 μL, and final cDNA product was kept in −20 °C for a few weeks preceding quantitative PCR. Expression of interferon regulatory factor 3 and 7 (*IRF-3*, *IRF-7*), tetherin (*BST2*), suppressor of cytokine signaling 1 and 3 (*SOCS1, SOCS3*), nuclear factor NF-kappa-B p105 and p65 subunit (*NFKB1*, *p65* alias *RELA*), and interferon-induced GTP-binding protein Mx1 (*MxA*) was studied by quantitative PCR (qPCR) using Taq DNA Polymerase with Sybr Green I dye. Data were normalized to endogenous reference gene *18S*, which was confirmed to be stable across the groups. Reaction was performed using SG qPCR Master Mix (EURx, Gdańsk, Poland) in combination with appropriate primers shown in Table 1. Furthermore, primer specificity was confirmed empirically after qPCR by performing melting curve analysis of every sample. All experiments were performed using Light Cycler 480 II instrument (Roche Diagnostics GmbH, Basel, Switzerland). Every reaction was performed two times and differences in quantification cycle between both repeats were negligible (±0.2). Quantification cycles (Cq) for every reaction were calculated automatically by the Light Cycler 480 II instrument software (Roche Diagnostics GmbH, Basel, Switzerland). Relative changes in mRNA expression were calculated using the delta Cq method with the healthy control as the comparator.

### 2.14. Statistical Analysis

To capture the central tendency (average) of the data, the Hodges–Lehman estimator—pseudo median—was used (in the following part called *Median*). Confidence intervals (CI95) at significance level α=0.05 were used to measure precision of estimation and testing some statistical hypothesis. CI95s were estimated with bootstrap method. Statistic $Sn=med\left\{med\left|x_{i}\;–\;x_{j}\right|;j=1\ldots n\right\}$ was used as robust measure of variability [19] as well as minimal and maximal observations. Sn is typical difference between two randomly sampled observations. Delta Δ is Hodges–Lehman estimator of the shift parameter between distributions of two independent populations. Delta Δ is median of differences between all pairs of observations, where one observation belongs to group A, and the second observation in the pair belongs to group B. Delta is shift parameter used also in Wilcoxon rank-sum test to comparison two independent populations (also known as Mann–Whitney test). Delta, CI95 for delta, and *p*-values of the delta were estimated numerically with bootstrap method because of a small sample sizes. In the case of investigation of an influence of EGb treatment on the level of innate immunity of AD patients and controls, an analysis of variance (ANOVA) was used. In the case of ANOVA, statistical effect size for every variable in the model was measured with partial eta-squared defined as $\eta^{2}=\frac{SS_{variable}}{SS_{variable}+SS_{error}}$, where $SS_{variable}$ and $SS_{error}\;$is sum of squares in ANOVA for effect of the variable and sum of squares for the experimental error, respectively. Interpretation of the $\eta^{2}$ was typical, after Cohen.

For every investigated i−th individual from AD patients and from controls, the effect of EGb treatment was calculated as $d_{i}=log_{e}\frac{level\;after\;treatment}{level\;before\;treatment}$ for all cytokine production and for all gene expression. An average effect in the group was estimated as pseudo median (*Median*) with Hodges–Lehman estimator (HL)f central tendency, so EGb effect=HL(d).

## 3. Results

### 3.1. Blood Donors (Study Groups)

Table 2 presents basic characteristics of 39 investigated blood donors—AD patients and controls (age-matched, over 55 years old)—according to a few variables. There were 22—15 female and 7 male—among AD patients and 17—10 female and 7 male—among controls. MMSE score was evaluated. MMSE median for female was 18.75 and for male 18.5. Among the AD group, there were no differences between men and women in the level of dementia. Patients were collected randomly to estimate DGN and DSMV differences between sex. Chi-square test statistic $\chi_{df=2}^{2}=0.598,\;p=0.856$.

### 3.2. EGb Characteristic (Cytotoxicity, Antioxidant Activity)

First, the starting solution of EGb was prepared. EGb at 20 mg/mL in DMSO was diluted to final concentrations of 25–500 μg/mL in RPMI 2% FBS. Freshly isolated PBLs were treated with several concentrations of EGb and incubated in 37 °C/5% CO_2_ for 24 h. After that, time morphological changes of the cells were observed under the inverted microscope. Total number and viability of PBLs were determined by 0.4% trypan blue staining. The viable cells—with intact cell membranes—did not take up impermeable trypan blue (stayed non-colored), whereas dead cells (with damaged cell membranes, cell shadows, shrunken cells) were permeable and took up the dye (dyed with distinctive blue). Fresh EGb dilutions were prepared before each experiment. Experiments was performed three times in two independent repetitions each. It was noticed that EGb in concentration over 200 μg/mL resulted in cytotoxicity (cell viability below 90%). EGb in the range of 25–150 µg/mL was nontoxic for PBLs (cell viability over 90%). Control were PBLs incubated only with culture medium RPMI 2% FBS. The final concentrations of DMSO < 2% were nontoxic. Estimated cytotoxic concentration of EGb, which reduced viability of PBLs by 50% (CC_50_), was calculated and equal to$\;CC_{50}≅743.5\;µg/\mathrm{mL}$. Based on cytotoxicity test for the future experiments of an influence of the extract on innate immune response of PBLs, the highest nontoxic concentration of EGb, − 150 µg/mL, was used. The cell viability for this concentration was about 95%.

An antioxidant activity (in vitro), i.e., ferric ion reducing antioxidant power, Fe^2+^ chelating ability, and DPPH radical scavenging activity of EGb were analyzed (Table 3). EGb preparation exerted strong DPPH scavenging activity; only 10 µg of preparation has the same effect as 0.03 µM Trolox. The antioxidant properties (inhibition concentration, IC_50_) for DPPH scavenging activity reached the value 41.13 µg. EGb preparation also exerted strong concentration-dependent ferric ion reducing antioxidant power and Fe^2+^ chelating ability (Table 3).

### 3.3. EGb Improves Innate Immune Response of PBLs

**Figure 1:**
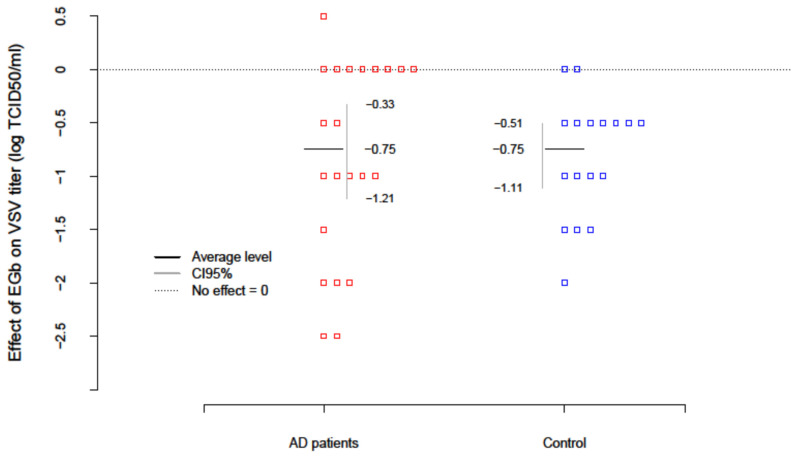
EGb effect on VSV replication (level of innate immunity) in PBLs from AD patients and healthy age-matched controls. EGb effect was measured as difference between VSV titer (log TCID_50_/mL) after EGb treatment and VSV titer (log TCID_50_/mL) before EGb treatment. Red and blue squares are individual observations.

The level of innate immunity of AD patients and healthy age-matched controls was estimated based on the test with vesicular stomatitis virus (VSV) replication in freshly isolated PBLs ex vivo. To evaluate the effect of EGb (150 µg/mL) on innate immunity VSV titers were examined after EGb treatment in the collected supernatants. Results are presented in Table 4. The level of innate immunity/PBLs resistance to VSV infection was assessed with the scale: the lack of virus replication (0–1 log TCID_50_/mL) indicated complete immunity; VSV replication over 1 log (about 2–3 log) indicated deficiencies and partial immunity; and VSV replication over 4 log evidenced high deficiency in innate immunity. The EGb effect in every patient was calculated as a difference between the level of innate immunity after EGb treatment and before EGb treatment (EGb effect = after−before). The average change of innate immunity in AD patients was −0.75; i.e., EGb increased the level of innate immunity of AD. The results were significant (p=0.0002), with confidence interval CI95(-Inf; −0.41). In the control group an increase in innate immunity (p=0.0001) was also observed. There was no difference, however, in the effect of EGb treatment between AD and controls (p=7539). In summary, EGb expressed a beneficial effect on innate immune response of PBLs. EGb treatment significantly increased the level of innate immunity in AD patients as well as controls (Figure 1).

**Figure 2:**
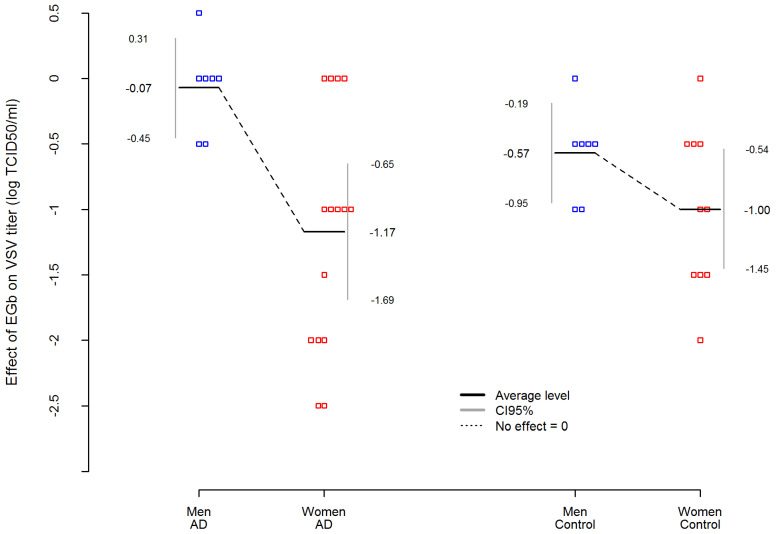
Sex-dependent differences in EGb effect on VSV replication/level of innate immunity in AD patients and healthy age-matched controls. EGb effect was measured as difference between VSV titer (log TCID_50_/mL) after EGb treatment and VSV titer (log TCID_50_/mL) before EGb treatment. Red and blue squares are individual observations.

In addition, Table 4 presents an analysis of variance (ANOVA) where EGb effect was dependent variable in AD and control group. The analysis showed that among AD patients, the effect of EGb treatment on the level of innate immunity was not related to the severity of the disease. Interestingly, an important variable was sex. The statistic effect size here was partial eta-squared η^2^ equals for sex effect η_Sex^2^ = 0.3983, which is commonly interpreted as a huge effect size according to Cohen’s interpretation. Thus, EGb increased the level of innate immunity much stronger in women with AD than in men with AD (p=0.0049). Simultaneously, the sex differences in EGb effect was smaller and not statistically significant in the control group (p=0.1883). The difference between AD women and AD men was more than 5.5 times higher than between women and men in the control group. Results are presented in Figure 2.

### 3.4. Immunoregulatory Effect of EGb Treatment on Cytokine Production by PBLs

In the light of obtained results of beneficial effect of EGb on the PBLs resistance to viral infection/level of innate immunity, it became interesting to investigate the impact of the extract on cytokine production, which is engaged in innate immune response, as changes in viral replication are related to changes in cytokine balance produced by whole PBLs. Table 5 shows an average level of cytokine production, namely TNF-α, IFN-γ, IL-10, IL-1β, and IL-15, by uninfected (spontaneous release) and VSV-infected PBLs from AD patients and controls. The effect of EGb treatment is also presented. As shown in Table 5, VSV infection of PBLs resulted in high TNF-α but slight IL-1β and IL-15 production in both groups. PBLs infection with VSV also revealed increased IFN-γ release but only in the control group. In the case of IL-10, VSV infection resulted in decreased production of this cytokine in AD patients and controls. Interestingly, EGb treatment differentially influenced on all investigated cytokines.

EGb decreased **TNF-α** production by uninfected (spontaneous) and VSV-infected PBLs from AD patients and controls. Notably, this effect was considerably higher in AD patients. The average level of spontaneous TNF-α production by PBLs from AD patients was $Med_{TNF-\alpha ;AD}^{PBLs;\;before}=27.17\;\mathrm{pg}/\mathrm{mL}$, and after EGb treatment, it decreased to $\;Med_{TNF-\alpha ;AD}^{PBLs;\;after}=1.01\;\mathrm{pg}/\mathrm{mL}$. This change (effect) was statistically significant at α=0.05. In the control group, the average level of spontaneous TNF-α production by PBLs before EGb treatment was $Med_{TNF-\alpha ;Control}^{PBLs;\;before}=49.02\;\mathrm{pg}/\mathrm{mL},$ and after EGb treatment, it decreased to $Med_{TNF-\alpha ;Control}^{PBLs;\;after}=17.2\;\mathrm{pg}/\mathrm{mL}$. This change (effect) was also statistically significant. There was significant difference in this effect between AD patients and controls. The decrease of spontaneous TNF-α production by PBLs from AD patients after EGb treatment was higher than in the control group (p=0.0107). Similarly, the average level of TNF-α produced by VSV-infected PBLs from AD was $Med_{TNF-\alpha ;AD}^{PBLs+VSV;\;before}=60.47\;\mathrm{pg}/\mathrm{mL},$ and after EGb treatment, it decreased to $Med_{TNF-\alpha ;AD}^{PBLs+VSV;\;after}=1.1\;\mathrm{pg}/\mathrm{mL}$. This change (effect) was statistically significant at α=0.05. For control group, the average level of TNF-α produced by VSV-infected PBLs was $Med_{TNF-\alpha ;Control}^{PBLs+VSV;\;before}=212.83\;\mathrm{pg}/\mathrm{mL}$, and after EGb treatment, it decreased to $Med_{TNF-\alpha ;Control}^{PBLs+VSV;\;after}=22.56\;\mathrm{pg}/\mathrm{mL}$. This change (effect) was also statistically significant at α=0.05. There was significant difference in this effect between AD patients and controls. The decrease of TNF-α produced by VSV-infected PBLs from AD patients after EGb treatment was higher than in the control group (p=0.0317).

In the case of **IFN-γ**, we can thereby see that EGb decreased spontaneous production of this cytokine in AD group from $Med_{INF-\gamma -\alpha ;AD}^{PBLs;\;before}=9.84\;\mathrm{pg}/\mathrm{mL}$ to $Med_{IFN-\gamma -\alpha ;AD}^{PBLs;\;after}=2.69\;\mathrm{pg}/\mathrm{mL}$. The change (effect) was statistically significant at α=0.05. This effect was not observed in the control group probably due to a small sample size. Thus, there was significant difference in EGb effect between AD patients and controls.The decrease of spontaneous INF-γ production in AD patients after EGb treatment was higher than in the control group (p=0.0003). Similarly, after EGb treatment, the decrease in IFN-γ production by VSV-infected PBLs from AD patients was observed—from $Med_{IFN-\gamma -\alpha ;AD}^{PBLs+VSV;\;before}=9.86\;\mathrm{pg}/\mathrm{mL}$ to $Med_{IFN-\gamma -\alpha ;AD}^{PBLs+VSV;\;after}=2.83\;\mathrm{pg}/\mathrm{mL}$. The change (effect) was statistically significant at α=0.05. For control group, the average level of INF-γ produced by VSV-infected PBLs was $Med_{IFN-\gamma -\alpha ;Control}^{PBLs+VSV;\;before}=8.66\;\mathrm{pg}/\mathrm{mL}$ and after EGb treatment, it decreased to $Med_{IFN-\gamma -\alpha ;Control}^{PBLs+VSV;\;after}=5.05\;\mathrm{pg}/\mathrm{mL}$. The change (effect) was also statistically significant at α=0.05. Significant difference in EGb effect between AD patients and controls was noticed. A decrease of INF-γ production by VSV-infected PBLs in AD patients after EGb treatment was higher than in the control group (p=0.0127).

The reducing effect of EGb treatment was shown either for anti-inflammatory cytokine **IL-10** production. EGb decreased spontaneous IL-10 production in AD group from $Med_{IL-10-\alpha ;AD}^{PBLs;\;before}=86.13\;\mathrm{pg}/\mathrm{mL}$ to $Med_{IL-10-\alpha ;AD}^{PBLs;\;after}=10.47\;\mathrm{pg}/\mathrm{mL}$. This change was statistically significant. In the control group, EGb decreased IL-10 production from $Med_{IL-10-\alpha ;AD}^{PBLs;\;before}=162.87\;\mathrm{pg}/\mathrm{mL}$ to $Med_{IL-10-\alpha ;AD}^{PBLs;\;after}=28.76\;\mathrm{pg}/\mathrm{mL}$. This change was also statistically significant. In the case of IL-10 production after VSV-infection, EGb decreased the level of this cytokine in AD patients from $\;Med_{IL-10-\alpha ;Control}^{PBLs+VSV;\;before}=43.49\;\mathrm{pg}/\mathrm{mL}$ to $Med_{IL-10-\alpha ;Control}^{PBLs+VSV;\;after}=2.37\;\mathrm{pg}/\mathrm{mL}$. This change was statistically significant. In the control group, EGb also decreased IL-10 production from $Med_{IL-10-\alpha ;Control}^{PBLs+VSV;\;before}=163.73\;\mathrm{pg}/\mathrm{mL}$ to $Med_{IL-10-\alpha ;Control}^{PBLs+VSV;\;after}=20.11\;\mathrm{pg}/\mathrm{mL}$. This change was also statistically significant. A decrease of IL-10 by uninfected and VSV-infected PBLs after EGb treatment was the same in AD patients and controls.

In contrast, EGb increased IL-1β and IL-15 production by uninfected and VSV-infected PBLs from AD and controls. EGb increased **IL-1β** production by uninfected PBLs from control group. The average level of spontaneous IL-1β production was $Med_{IL1-\beta -\alpha ;AD}^{PBLs;\;before}=288.6\;\mathrm{pg}/\mathrm{mL},$ and after EGb treatment, it was $\;Med_{IL-1\beta -\alpha ;AD}^{PBLs;\;after}=496.12\;\mathrm{pg}/\mathrm{mL}$. The change (effect) was statistically significant at α=0.05. This effect was not observed in AD group probably due to a small sample size. Similarly, the average level of IL-1β produced by VSV-infected PBLs from AD was $Med_{IL-1\beta -\alpha ;AD}^{PBLs+VSV;\;before}=401.33\;\mathrm{pg}/\mathrm{mL},$ and after EGb treatment, it increased to $Med_{IL-1\beta -\alpha ;AD}^{PBLs+VSV;\;after}=427.88\;\mathrm{pg}/\mathrm{mL}$. For control group, the average level of IL-1β produced by VSV-infected PBLs was $Med_{IL-1\beta -\alpha ;Control}^{PBLs+VSV;\;before}=550.54\;\mathrm{pg}/\mathrm{mL}$ and after EGb treatment, it increased to $Med_{IL-1\beta -\alpha ;Control}^{PBLs+VSV;\;after}=604.51\;\mathrm{pg}/\mathrm{mL}$. An increase of IL-1β by VSV-infected PBLs after EGb treatment was the same in AD patients and controls.

EGb treatment influenced positively and statistically significant on **IL-15** production by uninfected PBLs from AD and controls. An increase of this cytokine was observed, respectively, in AD from $Med_{IL-15-\alpha ;AD}^{PBLs;\;before}=22.7\;\mathrm{pg}/\mathrm{mL}$ to $\;Med_{IL-15-\alpha ;AD}^{PBLs;\;after}=39.59\;\mathrm{pg}/\mathrm{mL}$ and in controls from $Med_{IL-15-\alpha ;AD}^{PBLs;\;before}=30.79\;\mathrm{pg}/\mathrm{mL}$ to $Med_{IL-15-\alpha ;AD}^{PBLs;\;after}=42.96\;\mathrm{pg}/\mathrm{mL}$. In the case of IL-15 production by VSV-infected PBLs, an increased effect of EGb treatment was noticed, respectively, in AD from $Med_{IL-15-\alpha ;Control}^{PBLs+VSV;\;before}=23.7\;\mathrm{pg}/\mathrm{mL}$ to $Med_{IL-15-\alpha ;Control}^{PBLs+VSV;\;after}=39.11\;\mathrm{pg}/\mathrm{mL}$ and in controls from $Med_{IL-15-\alpha ;Control}^{PBLs+VSV;\;before}=40.71\;\mathrm{pg}/\mathrm{mL}$ to $Med_{IL-15-\alpha ;Control}^{PBLs+VSV;\;after}=48.62\;\mathrm{pg}/\mathrm{mL}$. An increase of IL-15 by uninfected and VSV-infected PBLs after EGb treatment was the same in AD patients and controls.

Additionally, the level of **IFN-α** was also investigated. Results showed that PBLs from AD and controls did not produce spontaneous IFN-α, which was expected. In every investigated person in both groups, spontaneous IFN-α production was undetectable. Infection with VSV resulted in high IFN-α production by PBLs in both groups. However, leukocytes from healthy age-matched subjects produced $Med_{IFN-\alpha ;Control}^{PBLs+VSV;\;before}=88.47\;\mathrm{pg}/\mathrm{mL}$—over eight times more IFN-α than leukocytes from AD patients $Med_{IFN-\alpha ;AD}^{PBLs+VSV;\;before}=10.49\;\mathrm{pg}/\mathrm{mL}$. EGb treatment decreased IFN-α, and this effect was very strong, to an undetectable level, in the case of all AD patients and controls. Obtained results were very interesting; however, data were not included in the Table 5 due to experiments that were performed only for half of the participants and need to be confirmed in a larger sample size.

**Figure 3:**
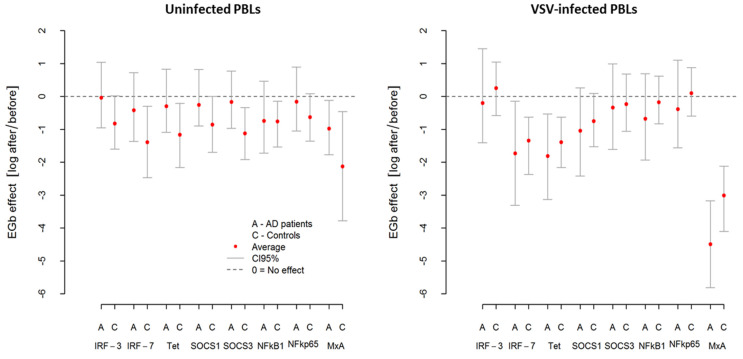
Influence of EGb on pro- and anti-inflammatory cytokine production by uninfected (spontaneous) and VSV-infected PBLs from AD patients and healthy age-matched controls. EGb effect was measured as natural logarithm of ratio after/before cytokine level. Zero means no effect. Points represent average change after treatment with confidence interval CI95% for the average.

In summary, EGb showed immunoregulatory activity resulted in a different influence on selected pro- and anti-inflammatory cytokine production by PBLs. It decreased TNF-α, IFN-γ, and IL-10 production but increased IL-1β and IL-15 production by uninfected and VSV-infected leukocytes of AD patients and controls. The effects of EGb treatment, however, were much stronger in AD patients. Results of EGb treatment on cytokine production are presented in Figure 3.

### 3.5. EGb Treatment Down-Regulates Inflammatory-Associated Genes Expression

To detect and respond to viral infection host cell activates multiple cellular signaling networks. Viral proteins and nucleic acids ultimately drive an antiviral response and activating transcription factors. Therefore, to study the influence of EGb on innate immune mechanisms (PBLs resistance to VSV infection/level of innate immunity and cytokine production), we investigated the expression of *IRFs-3* and -*7* (interferon regulatory factors) mRNA as well as ISGs (IFN-stimulated genes)—*tetherin* (encoding bone marrow stromal cell antigen 2) and *MxA* (encoding myxovirus resistance protein 1). *NF-κB* transcription factors and *SOCS* (suppressor of the cytokine signaling proteins) were also examined.

**Figure 4:**
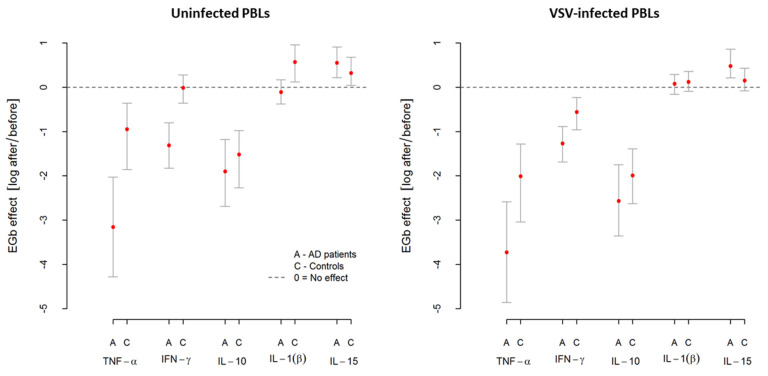
Changes in relative expression of inflammatory-associated genes after EGb treatment in uninfected and VSV-infected PBLs from AD patients and healthy age-matched controls. EGb effect was measured as natural logarithm of ratio after/before gene expression. Zero means no effect. Points represent average change after treatment with confidence interval CI95% for the average.

As was suspected, VSV infection resulted in upregulation in all investigated genes in AD patients and controls. However, this effect was more pronounced in AD patients. From Table 6, we can see that *MxA* was the most highly expressed gene with over 40 relative fold change in AD, while in controls, it was over 16 relative fold change. Similarly, after VSV infection in AD, a marked induction (3–8.5 relative fold change) of *IRF-7*, *tetherin,* and *SOCS1* was demonstrated. As presented in Figure 4 and Table 6, in both groups (AD and controls), EGb treatment decreased expression of all investigated genes (average effect < 0) in uninfected and VSV-infected PBLs. In uninfected PBLs from AD patients, average reduction in all investigated genes expression after EGb treatment was about $EGb\;effect_{all\;genes;AD}^{PBLs}\approx -0.4;$ i.e., the average level of expression was $\frac{1}{e^{-0.4}}=1.5\;\mathrm{times}\;$lower compared to the expression before EGb treatment. In the case of controls, it was $EGb\;effect_{all\;genes;Controls}^{PBLs}\approx -1.1;\;$i.e., average reduction in genes expression in controls after EGb treatment was $\frac{1}{e^{-1.1}}=3\;\mathrm{times}\;\mathrm{lower}$ compared to the expression before EGb treatment. Interestingly, in VSV-infected PBLs the effect of EGb treatment was stronger in AD group for all genes than for controls. In PBLs from AD patients, average reduction of all investigated genes expression was about $EGb\;effect_{all\;genes;AD}^{PBLs+VSV}\approx -1;\;i$ i.e., the average level of expression was $\frac{1}{e^{-1}}=2.7$ times lower compared, to the genes expression before EGb treatment. In PBLs from control group it was $EGb\;effect_{all\;genes;Controls}^{PBLs+VSV}\approx -0.7;$ i.e., the average expression in VSV-infected leukocytes was $\frac{1}{e^{-0.7}}=2\;$times lower compared to genes expression before EGb treatment. Table 6 presents level of investigated genes expressions before and after EGb treatment, EGb effects, and comparison of EGb effects in both groups. Confidence intervals (CI95) in Figure 4 and in Table 6 show greater precision of expression measurement than testing for statistical significance.

## 4. Discussion

With numbers of sufferers with cognitive impairments still increasing, dementia such as Alzheimer’s disease (AD) is observed. AD is an age-related disorder; however, neurodegenerative changes may begin many years before clinical manifestation of the disease. Therefore, brain health is becoming very important for the adults and the elderly. The lack of effective AD treatment means that those affected by this disease are still looking for any alternative approaches. Nutritional science and dietary supplements, such as extracts of *Ginkgo biloba* (EGb), offer an approach (preventative and restorative) to adding phytonutrients to daily consumption of food and drink [20]. EGb is the most widely studied natural compound with proven beneficial effect on cognitive functions (improving memory and concentration) in both healthy adults and patients with mild cognitive impairment (MCI) or dementia. However, its effect on the immune system is less explored, with little literature data available. We have previously reported that EGb increased an innate immune response and regulated cytokine production in healthy young people [21,22]. We suggest the possibility of using EGb for the treatment of immune deficiencies. As AD is considered as a systemic disease with evidence for changes in immune system functioning, we examined the effect of EGb on innate immune mechanisms in AD patients. In the present study, we showed that EGb is a good immunoregulator increasing an innate immune response of AD patients. We also examined the effect of EGb treatment on several inflammatory-associated genes expression.

Currently, many EGb preparation are available, with different compositions resulting in various therapeutic features [23]. We used a standardized extract, prepared according to the European Pharmacopoeia (Ph. Eur. 8.0); it is easily accessible, which is a great benefit. In addition, an advantage of herbal drugs is that they have very low side-effects compared to chemical drugs [6]. Indeed, we observed immuno-enhancing activity of EGb in an absolutely nontoxic concentration for human peripheral blood leukocytes (PBLs). The leaves of maidenhair tree (*G. biloba*) are also excellent sources of antioxidants. Ethanol extracts of that plant are well-characterized and contain about 22–27% flavonol glycosides, including polyphenols such as tannins and terpene lactones (5–7%). The high phenolics content determines strong ferric ion activity, reducing antioxidant power, copper chelating ability, peroxyl radical scavenging activity, and radical scavenging activity for *G. biloba* extracts [24,25,26]. Our results correspond to the results of a study on antioxidant properties of various plant extracts carried out by Szerlauth and others (2019) [24]. It was found that the plant extracts exhibit remarkable antioxidant properties (IC_50_) from 0.44 (*Alliumsativum*) to 44.18 (*Juglansregia*) AAEQ values [24].

The observed age-related progressive decline in immune system functioning contributes to a development of chronic states of inflammation resulting in systemic diseases, including AD. This phenomenon—dysregulation of the immune system—is not limited to the one mechanism, but it concerns aging of the innate and adaptive immune cells, alterations in circulating inflammatory mediators, and changes in lymphoid and non-lymphoid tissues [4]. In AD patients, dysregulation of innate immune mechanisms were also observed. PBLs resistance to viral infection is a good indicator of the innate immune system condition. We previously established that the level of innate immunity, measured with the test based on vesicular stomatitis virus (VSV) replication in human PBLs, was remarkably correlated with clinical advancement of AD. The higher VSV titer means a lower level of innate immunity. More severe patients were characterized with a lower level of innate immunity [27]. We also showed as a potential therapeutic the oral administration of proline-rich polypeptide complex (PRP) isolated from bovine colostrum, a nutraceutical intended to boost an immune system by increasing an innate immune response in AD patients [27]. Here, we present for a first time the strong immune-enhancement activity of natural, herbal preparation, EGb, in an ex vivo model of PBLs from AD patients. As we suspected, EGb was capable of significantly improving innate immune response by decreasing VSV replication in PBLs of AD patients but also controls. The most interesting, however, was that EGb notably increased an innate immune response in AD women. This sex discrepancy in EGb effect was not observed in the control group. Similarly, in the above-mentioned study of EGb effect on the immune system functioning in healthy young people, sex differences were not observed. Thus, EGb may be promising for immune improving in AD patients, especially in AD women. It is important due to the fact that women are more afflicted with the frequency, prevalence, and clinical manifestation of the disease [28].

According to recent data in animal models, EGb showed wide-ranging anti-inflammatory and antioxidant properties [29]. The immune effect of EGb was showed by Wan at al. [30] in the model of mouse microglia cells. Microglia, brain resident macrophages, play an important role in the development of many central nervous system (CNS) diseases. Reactive microglia that produce large amounts of inflammatory mediators influence the development of AD [31]. Significantly reduced production of pro-inflammatory cytokines, such as IL-6 and TNF-α, and increased anti-inflammatory IL-4, IL-13, or TGF-β were found in the brains of animals supplemented with EGb [30]. Similar results were presented by Tao et al. [32] in allergic mice. Administration of EGb showed significant decrease in release of pro-inflammatory IL-4, IL-5, IL-6, IL-8, and TNF-α. In our study with human leukocytes model ex vivo from AD patients and healthy age-matched controls, EGb presented immunoregulatory activity. EGb significantly decreased production of pro-inflammatory TNF-α and IFN-γ as well as anti-inflammatory IL-10 by uninfected and VSV-infected PBLs of AD patients and controls. At the same time, EGb significantly increased production of IL-15 and slightly increased IL-1β in both groups of participants. Interestingly, this effect was more pronounced among AD patients.

It was established that VSV infection of PBLs induced secretion of IFN-α [33]. In the present study, IFN-α was also investigated. IFN-α was not assessed for all study participants, and this investigation needs to be continued. However, interesting observations were obtained. Although, as suspected, there was no spontaneous secretion of IFN-α, after VSV infection, leukocytes of both groups responded with production of this cytokine. After incubation with EGb, the production of IFN-α decreased to no detectable level in all AD patients as well as in controls. IFN-α was the only cytokine strongly inhibited by the extract.

EGb also downregulated expression of several genes that regulate innate immune response to viral infection and cytokine production, such as interferon regulatory factors (IRFs) *IRF-3* and *-7*, which are primary transcriptional factors regulate the type I IFN response after RNA virus infections [34]; IFN-stimulated antiviral genes *MxA* and *tetherin*, critical for controlling VSV infection [33]; but also *NFĸB* transcription factors that mediate induction of various pro-inflammatory genes in innate immune cells; and *SOCS*, the main regulators of antimicrobial innate immune response [35,36]. It was presented earlier that *G. biloba* extract has anti-oxidative as well as anti-inflammatory properties. A marked suppression of transcription factor NFκB and pro-inflammatory cytokines (TNF-α, IL-1α, 1L-6) was shown [37].

## 5. Conclusions

Therefore, EGb may have an advantageous properties for health management in elderly and AD sufferers but especially in women afflicted with AD. Female sex is a major risk factor for developing late-onset AD, which is suggested to be implicated in the menopause transition [38]. The observed beneficial effect of EGb on innate immune response/increase PBLs resistance to VSV infection may be at least partially explained by its antioxidant activity and differential influence on cytokine production. Even though the most important antiviral response is mediated with IFN I, the role of other cytokines should not be diminished. In our study, EGb decreased IFNs production but increased IL-15 and IL-1β. IL-15 is known as playing an important role in promoting the development and homeostasis of NK cells and CD8 T; however, IL-15 also mediates the anti-viral responses of these cell populations during an active immune response [39]. IL-1β, next to type I IFNs, is also a central mediator driving innate antiviral immunity and inflammation [40]. It was suggested that activation status of peripheral innate immune cells may be a good biomarker of AD pathology [3]. Thus, improving their activity by adding EGb as accompanying treatment may be a good long-term course to modify the disease progression.

## Figures and Tables

**Table 1:** Primers used for real-time PCR.

Gene	Primer Sequence	
*IRF-3* [15]	F1	5′-ACC ACC CGT GGA CCA AGA G-3′
	R1	5′-TAC CAA GGC CCT GAG GCA C-3′
*IRF-7* [15]	F2	5′-TGG TCC TGG TGA AGC TGG AA-3′
	R2	5′-GAT GTC ATA GAG GCT GTT GG-3′
*BST2* [15]	F3	5′-AAG AAA GTG GAG CTT GAG G-3′
	R3	5′-CCT GGT TTT CTC TTC TCA GTC G-3′
*SOCS1* [16]	F4	5′-GGA ACT GCT TTT TCG CCC TTA-3′
	R4	5′-AGC AGC TCG AAG AGG CAG TC-3′
*SOCS3* [16]	F5	5′-CAA GGA CGG AGA CTT CGA TT-3′
	R5	5′-GGA GCC AGC CTG GAT CTG-3′
*NFKB1* [17]	F6	5′-AGA AGT CTT ACC CTC AGG TCA-3′
	R6	5′-CAG TTA CAG TGC AGA TCC CA-3′
*p65* [17]	F7	5′-GAA TGG CTC GTC TGT AGT GC-3′
	R7	5′-GCT GCT CAA TGA TCT CAA CAT-3′
*MxA* [15]	F8	5′-GCC GGC TGT GGA TAT GCT A-3′
	R8	5′-TTT ATC GAA ACA TCT GTG AAA GCA A-3′
*18S* [18]	F9	5′-GAA TGG CTC ATT AAA TCA GTT ATG G-3′
	R9	5′-TAT TAG CTC TAG AAT TAC CAC AGT TAT CC-3′

**Table 2:** Characteristics of blood donors—AD patients and controls.

Variable	Group	Median	Sn	Min–Max
Age	AD	68	11	32–80
	Control	61.5	9.5	42–90
Sex	**Group**	**Women**	**Men**	**% Men**
	AD	15	7	31.8
	Control	10	7	41.2
MMSE	**AD Group**	**Median**	**Sn**	**Min–Max**
	Women	18.75	3	12–24
	Men	18.5	3.5	12–23
DGN	**AD Group**	**Mild**	**Moderate**	**Serious**
	Women	4	6	5
	%	26.7	40.0	33.3
	Men	3	2	2
	%	42.9	28.6	28.6
DSMV	**AD Group**	**Mild**	**Moderate**	**Serious**
	Women	4	6	5
	%	26.7	40.0	33.3
	Men	3	2	2
	%	42.9	28.6	28.6

Sn, measure of variability; MMSE, mini-mental state examination; DGN, the diagnostic according to the guidelines of the German Society of Neurology; DSMV, fifth edition of diagnostic and statistical manual of mental disorders classification (criteria for major neurocognitive disorder).

**Table 3:** Concentration-dependent antioxidant activity (in vitro) of EGb.

EGb	DPPH		FRAP	Chelation
(µg)	(µMTrolox_eq_)	(%) Inactivated DPPH	(µg Fe^2+^)	(µg Fe^2+^)
250	or	or	or	62.63
150	or	or	or	43.82
100	or	or	or	24.68
50	0.09	63.51	17.24	20.2
10	0.03	23.31	4.65	14.68
5	0.02	12.73	2.74	2.7

**Table 4:** Influence of EGb treatment on PBLs resistance/level of innate immunity in AD patients and healthy age-matched controls (*n* = 39).

PBLs Resistance/Level of Innate Immunity	Group	Median	Sn	Min; Max	Δ	*p*-Value
EGb effect	AD	−0.75	1	−2.5; 0.5	−0.75 CI95(−Inf; −0.41)	0.0002 ^1^
	Control	−0.75	0.5	−2; 0	−0.75 CI95(−Inf; −0.55)	0.0001 ^1^
	AD vs. Control				0.00 CI95(−0.49; 0.61)	0.7539
	ANOVA in AD group EGb effect depending on sex, DGN, MMSE, and age					
	**Variable**	$\mathrm{Effect size}\;\eta {\;}^{2}$ **(interpret.)**			$F_{df=1;16}$	***p*-Value**
	Sex $\beta_{women}=-1.18$	0.3983 (large)			10.59	0.0049
	DGN (=DSMV)	0.0503 (small/medium)			0.42	0.6616
	Age	0.0026 (negligible)			0.042	0.8406
	MMSE	0.0000 (no effect)			0.0001	0.9923
	ANOVA in Control group Effect of EGb treatment depending on sex and age					
	**Variable**	$\mathrm{Effect size}\;\eta {\;}^{2}$ **(interpret.)**			$F_{df=1;14}$	***p*-Value**
	Sex $\beta_{woman}=-0.21$	0.1202 (medium)			1.91	0.1883
	Age	0.0963 (medium)			1.49	0.2420

Δ, shift parameter of location (as a part of Wilcoxon rank test, Hodges-Lehman estimator of (pseudo) median); Sn, measure of variability (higher value means higher variability; robust estimator equivalent of standard deviation); ^1^, one side (left side) test and one side confidence interval; ^2^, Inf—infinity; $\;\beta_{woman}$, standardized difference between men and women adjusted with other variables present in ANOVA; MMSE, mini-mental state examination; DGN, the diagnostic according to the guidelines of the German Society of Neurology; DSMV, fifth edition of diagnostic and statistical manual of mental disorders classification (criteria for major neurocognitive disorder).

**Table 5:** Uninfected (spontaneous) and VSV-infected cytokine production by PBLs. Effect of EGb treatment. Median is measure of average level. For every individual person and for every change in cytokine level after EGb, treatment equals $\;d=\log\frac{after}{before}=\log\left(after\right)-\log\left(before\right)$. EGb effect is average of all d's in a group. EGb effect is than average of ratios $\frac{after}{before}$ expressed in logs. Delta Δ is measure of difference in EGb effects between AD and controls, and this statistic is the average difference between a randomly selected person from AD group and a randomly selected person from control group.

		Uninfected PBLs			VSV-Infected PBLs		
TNF−α		PBLs	PBLs + EGb	EGb effect	PBLs + VSV	PBLs + VSV + EGb	EGb effect
AD	Median	27.17	1.01	−3.16	60.47	1.1	−3.73
	CI95	9.55; 79.21	0.23; 3.54	−4.28; −2.03	19.58; 153.66	0.31; 3.74	−4.86; −2.59
Controls	Median	49.02	17.2	−0.95	212.83	22.56	−2.01
	CI95	24.88; 99.64	6.49; 41.73	−1.86; −0.36	100.85; 445.43	8.1; 63.35	−3.04; −1.28
AD vs. Controls	Δ	−8.14	−13.41	−1.96	−151.6	−20.93	−1.51
	CI95	−49.23; 31.88	−43.47; −2.09	−3.41; −0.56	−420.02; −7.61	−76.2; −2.35	−3.28; −0.06
	*p*-value (EGb effect AD vs. controls) = 0.0107				*p*-value (EGb effect AD vs. controls) = 0.0317		
**IFN−γ**		**PBLs**	**PBLs + EGb**	**EGb effect**	**PBLs + VSV**	**PBLs + VSV + EGb**	**EGb effect**
AD	Median	9.84	2.69	−1.31	9.86	2.83	−1.27
	CI95	6.84; 14.24	1.75; 4.28	−1.83; −0.8	6.95; 12.88	1.87; 4.21	−1.69; −0.89
Control	Median	3.29	3.31	−0.01	8.66	5.05	−0.56
	CI95	1.84; 5.08	1.69; 6.06	−0.36; 0.28	5.02; 13.68	3.09; 7.07	−0.96; −0.23
AD vs. Controls	Δ	6.03	−0.72	−1.28	0.45	−2.35	−0.69
	CI95	3.48; 9.41	−3.38; 1.17	−1.95; −0.55	−4.17; 4.83	−4.21; −0.21	−1.2; −0.15
	*p*-value (EGb effect AD vs. controls) = 0.0003				*p*-value (EGb effect AD vs. controls) = 0.0127		
**IL−10**		**PBLs**	**PBLs + EGb**	**EGb effect**	**PBLs + VSV**	**PBLs + VSV + EGb**	**EGB effect**
AD	Median	86.13	10.47	−1.9	43.49	2.37	−2.57
	CI95	34.77; 232.97	3.51; 34.75	−2.69; −1.18	15.73; 108.76	1; 6.58	−3.36; −1.75
Control	Median	162.87	28.76	−1.52	163.73	20.11	−1.99
	CI95	60.29; 356.65	10.13; 110.43	−2.27; −0.98	70.42; 369.71	8.93; 46.73	−2.63; −1.39
AD vs. Control	Δ	−70.75	−15.99	−0.26	−150.13	−17.55	−0.51
	CI95	−338.92; 57.65	−94.28; 14.73	−1.07; 0.52	−315.32; −13.16	−48.42; −2.18	−1.53; 0.41
	*p*-value (EGb effect AD vs. controls) = 0.5322				*p*-value (EGb effect AD vs. controls) = 0.3104		
**IL−1β**		**PBLs**	**PBLs + EGb**	**EGb effect**	**PBLs + VSV**	**PBLs + VSV + EGb**	**EGb effect**
AD	Median	367.64	328.83	−0.11	401.33	427.88	0.08
	CI95	238.78; 486.76	249.2; 424.48	−0.38; 0.17	302.81; 510.61	294.89; 552.79	−0.16; 0.29
Control	Median	288.6	496.12	0.57	550.54	604.51	0.12
	CI95	199.13; 399.27	329.64; 706.04	0.12; 0.96	403.5; 718.55	437.85; 830.44	−0.09; 0.36
AD vs. Control	Δ	73.16	−190.84	−0.71	−148.62	−180.34	−0.03
	CI95	−51.88; 211.25	−398.28; 0.32	−1.17; −0.2	−337.82; −6.34	−497.08; 34.26	−0.36; 0.26
	*p*-value (EGb effect AD vs. controls) = 0.005				*p*-value (EGb effect AD vs. controls) = 0.8316		
**IL−15**		**PBLs**	**PBLs + EGb**	**EGb effect**	**PBLs + VSV**	**PBLs + VSV + EGb**	**EGb effect**
AD	Median	22.7	39.59	0.55	23.7	39.11	0.48
	CI95	16.47; 31.27	30.9; 50.51	0.22; 0.91	18; 31.03	29.49; 54.57	0.21; 0.86
Control	Median	30.79	42.96	0.32	40.71	48.62	0.15
	CI95	23.05; 40.39	33.36; 56.07	0.04; 0.68	31.68; 54.52	35.66; 67.56	−0.08; 0.43
AD vs. Control	Δ	−8.58	−2.9	0.22	−15.44	−8.45	0.32
	CI95	−16.88; −0.29	−12.49; 5.47	−0.22; 0.69	−23.72; −9.17	−22.42; 3.89	−0.06; 0.69
	*p*-value (EGb effect AD vs. controls) = 0.3451				*p*-value (EGb effect AD vs. controls) = 0.0917		

**Table 6:** Genes expression before and after EGb treatment and measure of EGb effect in AD patients and control group. Median is measure of average level. For every individual person and for every gene change of expression level after, treatment equals $\;d=\log\frac{after}{before}=\log\left(after\right)-\log\left(before\right)$. EGb effect is average of all ds in a group. EGb effect is the average of ratios $\frac{after}{before}$ expressed in logs. Delta Δ is measure of difference in EGb effects between AD and controls, and this statistic is the average difference between a randomly selected person from AD group and a randomly selected person from control group.

		Uninfected PBLs			VSV-Infected PBLs		
IRF−3		PBLs	PBLs + EGb	EGb effect	PBLs + VSV	PBLs + VSV + EGb	EGB effect
AD	Median	1.65	1.78	−0.04	2.88	2.21	−0.2
	CI95	0.76; 4.01	0.59; 4.78	−0.95; 1.04	0.61; 9.7	1.04; 6.61	−1.41; 1.45
Control	Median	2.62	1.17	−0.82	1.95	2.53	0.25
	CI95	1.08; 6.3	0.61; 2.25	−1.6; 0.02	1.05; 3.73	0.98; 6.14	−0.58; 1.05
AD vs. Control	delta	−1.1	0.92	0.81	2.07	−0.18	−0.56
	CI95	−6.43; 1.22	−0.79; 3.75	−0.57; 2.13	−1.28; 9.98	−4.61; 6.35	−1.99; 1.37
	*p*-value	*p* = 0.2371			*p* = 0.4612		
**IRF−7**		**PBLs**	**PBLs + EGb**	**EGb effect**	**PBLs + VSV**	**PBLs + VSV + EGb**	**EGB effect**
AD	Median	1.39	1.06	−0.42	11.78	1.83	−1.73
	CI95	0.58; 4.08	0.36; 3.01	−1.37; 0.72	3.31; 32.97	0.67; 7.28	−3.31; −0.14
Control	Median	2.1	0.52	−1.39	7.84	2.1	−1.34
	CI95	0.74; 5.9	0.29; 0.96	−2.47; −0.3	4.44; 14.53	0.71; 5.13	−2.37; −0.63
AD vs. Control	delta	−1.06	0.77	1.02	7.81	0.18	−0.35
	CI95	−5.65; 2.38	−0.37; 3.19	−0.62; 2.54	−5.26; 39.16	−3.96; 16.53	−2.12; 1.79
	*p*-value	*p* = 0.1921			*p* = 0.7206		
**Tet**		**PBLs**	**PBLs + EGb**	**EGb effect**	**PBLs + VSV**	**PBLs + VSV + EGb**	**EGB effect**
AD	Median	1.32	1.12	−0.3	8.27	1.16	−1.81
	CI95	0.63; 2.94	0.38; 2.58	−1.09; 0.83	2.12; 24.23	0.58; 3.58	−3.13; −0.53
Control	Median	2.12	0.63	−1.16	6.66	1.79	−1.39
	CI95	0.85; 5.1	0.3; 1.28	−2.16; −0.21	3.89; 11.87	0.69; 4.59	−2.16; −0.63
AD vs. Control	delta	−1.23	0.59	0.88	3.76	−0.89	−0.43
	CI95	−5.11; 0.87	−0.44; 1.81	−0.53; 2.39	−5.16; 19.04	−4.57; 3.69	−2.12; 1.26
	*p*-value	*p* = 0.2264			*p* = 0.5552		
**SOCS1**		**PBLs**	**PBLs + EGb**	**EGb effect**	**PBLs + VSV**	**PBLs + VSV + EGb**	**EGB effect**
AD	Median	3.51	3.09	−0.26	11.94	3.63	−1.04
	CI95	1.6; 8.11	1.2; 6.88	−0.9; 0.82	3.64; 32.57	1.54; 15.29	−2.42; 0.26
Control	Median	2.28	0.97	−0.86	4.57	2.37	−0.75
	CI95	0.94; 5.72	0.55; 1.68	−1.7; 0	2.89; 7.79	0.97; 5.17	−1.53; 0.09
AD vs. Control	delta	0.84	2.73	0.63	10.7	2.62	−0.3
	CI95	−2.86; 5.41	0.16; 6.75	−0.58; 1.89	−1.6; 34.36	−3.24; 36.4	−2; 1.48
	*p*-value	*p* = 0.2868			*p* = 0.6482		
**SOCS3**		**PBLs**	**PBLs + EGb**	**EGb effect**	**PBLs + VSV**	**PBLs + VSV + EGb**	**EGB effect**
AD	Median	2.52	2.34	−0.17	3.93	2.43	−0.34
	CI95	1.29; 5.7	0.86; 5.73	−0.97; 0.77	0.9; 14.48	1.09; 10.14	−1.61; 0.99
Control	Median	2.26	0.68	−1.12	1.29	1.22	−0.23
	CI95	0.87; 4.98	0.34; 1.38	−1.92; −0.34	0.81; 2.61	0.5; 2.78	−1.06; 0.68
AD vs. Control	delta	−0.06	1.9	0.92	4.61	2	−0.17
	CI95	−2.96; 3.8	0.16; 5.99	−0.22; 2.17	−0.65; 17.48	−1.32; 23.93	−1.73; 1.56
	*p*-value	*p* = 0.1284			*p* = 0.8019		
**NFkB1**		**PBLs**	**PBLs + EGb**	**EGb effect**	**PBLs + VSV**	**PBLs + VSV + EGb**	**EGB effect**
AD	Median	2.99	1.63	−0.74	3.87	1.77	−0.68
	CI95	1.41; 7.73	0.55; 4.45	−1.72; 0.46	0.78; 15.27	0.79; 6.55	−1.93; 0.69
Control	Median	2.09	0.87	−0.76	1.88	1.75	−0.18
	CI95	0.99; 4.11	0.45; 1.72	−1.54; −0.14	1.14; 3.26	0.78; 3.82	−0.83; 0.62
AD vs. Control	delta	0.88	0.94	−0.01	5.12	0.5	−0.55
	CI95	−1.87; 7.59	−0.62; 4.33	−1.25; 1.56	−1.06; 21.44	−3.2; 11.06	−2.02; 1.13
	*p*-value	*p* = 0.9873			*p* = 0.4645		
**NFkp65**		**PBLs**	**PBLs + EGb**	**EGb effect**	**PBLs + VSV**	**PBLs + VSV + EGb**	**EGB effect**
AD	Median	2.63	2.5	−0.16	4.71	2.9	−0.39
	CI95	1.16; 6.81	0.92; 6.18	−1.05; 0.89	0.98; 16.35	1.38; 10.06	−1.56; 1.1
Control	Median	1.93	0.99	−0.63	2.06	2.46	0.1
	CI95	0.88; 4.11	0.56; 1.75	−1.36; 0.08	1.22; 3.46	1.01; 5.68	−0.6; 0.88
AD vs. Control	delta	0.77	1.9	0.42	5.28	0.79	−0.55
	CI95	−2.05; 6.57	−0.12; 5.4	−0.79; 1.84	−0.94; 20.13	−4.27; 17	−1.99; 1.17
	*p*-value	*p* = 0.5612			*p* = 0.4365		
**MxA**		**PBLs**	**PBLs + EGb**	**EGb effect**	**PBLs + VSV**	**PBLs + VSV + EGb**	**EGB effect**
AD	Median	0.47	0.2	−0.98	21.82	0.22	−4.49
	CI95	0.17; 1.32	0.07; 0.37	−1.77; −0.12	6.87; 64.67	0.1; 0.62	−5.81; −3.17
Control	Median	1.32	0.15	−2.13	21.69	1.07	−3.01
	CI95	0.27; 6.13	0.07; 0.32	−3.78; −0.46	14.08; 33.2	0.34; 3.57	−4.1; −2.12
AD vs. Control	delta	−2.23	0.02	1.19	6.3	−1.54	−1.5
	CI95	−8.97; 0.34	−0.42; 0.44	−1.14; 3.17	−17.07; 73.42	−3.86; 0.03	−3.15; 0.07
	*p*-value	*p* = 0.3098			*p* = 0.069		
